# Research Progress on Using Modified Hydrogel Coatings as Marine Antifouling Materials

**DOI:** 10.3390/md22120546

**Published:** 2024-12-03

**Authors:** Ying Wang, Xiaohong Zhou, Lingyan He, Xiangkai Zhou, Yantian Wang, Peijian Zhou

**Affiliations:** 1 College of Metrology Measurement and Instrument, China Jiliang University, Hangzhou 310018, China; 18255922629@163.com (Y.W.); s24020804085@cjlu.edu.cn (X.Z.); s24020804061@cjlu.edu.cn (Y.W.); zhoupj@cjlu.edu.cn (P.Z.); 2 School of Mathematics, Physics and Optoelectronic Engineering, Hubei University of Automotive Technology, Shiyan 442002, China; zxh-2233@163.com; 3 College of Mechanical and Electrical Engineering, Guangxi Vocational College of Water Resources and Electric Power, Nanning 530023, China

**Keywords:** modified hydrogel, hydrogel coating, marine antifouling, marine biofouling

## Abstract

The adhesion of marine organisms to marine facilities negatively impacts human productivity. This phenomenon, known as marine fouling, constitutes a serious issue in the marine equipment industry. It increases resistance for ships and their structures, which, in turn, raises fuel consumption and reduces ship speed. To date, numerous antifouling strategies have been researched to combat marine biofouling. However, a multitude of these resources face long-term usability issues due to various limitations, such as low adhesion quality, elevated costs, and inefficacy. Hydrogels, exhibiting properties akin to the slime layer on the skin of many aquatic creatures, possess a low frictional coefficient and a high rate of water absorbency and are extensively utilized in the marine antifouling field. This review discusses the recent progress regarding the application of hydrogels as an important marine antifouling material in recent years. It introduces the structure, properties, and classification of hydrogels; summarizes the current research status of improved hydrogels in detail; and analyzes the improvement in their antifouling properties and the prospects for their application in marine antifouling.

## 1. Introduction

In recent years, technological and scientific advances have paralleled the world’s exponential population growth, driving both economic and industrial development. The demand for resources has also increased, adding to the burden on natural resources. Despite the positive global economic impact of fisheries, maritime transportation, and offshore oil and gas, the challenges tied to marine technology, transportation infrastructure, and the offshore industry pose considerable impediments to transportation efficiency and production quality. Marine biofouling is one such complication and has become a serious global problem. Fouling organisms, such as macroalgae, barnacles, and shells, occur in abundance in nature, and these fouling organisms can attach to and colonize a wide variety of surfaces, including ships, pipelines, and the like. Marine organism fouling can result in numerous detrimental effects, notably increased navigation resistance, reduced ship speed, escalated fuel consumption, and an overall dip in ships’ service performance [1,2,3]. Marine biofouling on the surfaces of offshore products typically commences with the formation of micron-scale biofilms on the product’s surface, in turn, enticing both microscopic and macroscopic aquatic organisms to adhere. The substances excreted through the growth of attached organisms corrode and form constant deposits on the surface of the material, culminating in reduced equipment lifespan, increased vessel navigational resistance, and other complications; these effects all contribute to substantial losses across maritime and offshore ventures [4,5]. Marine biofouling prevention strategies either target the early stages by avoiding the formation of organic/microbial films on the material’s surface or aim at eliminating or dislodging entrenched fouling organisms in the latter stages [6].

Hydrogels, as polymers, feature a low coefficient of friction, a three-dimensional network structure, and commendable water absorption properties [7]. Antifouling coatings, synthesized from hydrogel materials and applied on marine infrastructures, exhibit the notable property of preventing marine organism adhesion [8,9,10]. Furthermore, hydrogel antifouling coatings possess a myriad of distinctive characteristics that are typically absent in other antifouling coatings (for example, hydrophobic antifouling coatings). These unique properties include a low surface energy, non-toxicity, environmental friendliness, as well as high levels of smoothness and softness [11,12,13,14,15,16].

Research into the enhancement of hydrogels is presently a central focus in the field of marine antifouling [17]. Hydrogel coatings based on polyvinyl alcohol (PVA) exhibit commendable biocompatibility and water solubility, thereby effectively impeding bio-adhesion and pollution [18]. However, they fall short in terms of durability and mechanical strength. It would be worthwhile to explore the alterations to cross-linking methods and mechanical properties to bolster durability. Coatings made from polyethylene glycol (PEG) hydrogel demonstrate superb hydrophilicity and minimal protein adsorption, which mitigates bio-adhesion and reduces protein pollution. Despite their durability being marginally inferior to that of other varieties, this can be counteracted through the employment of hybridized network structures and other such methodologies. Hydrogel–organic resin hybridized network coatings amalgamate the benefits of both hydrogels and organic resin, leading to good weathering, antifouling, and mechanical properties in marine environments. Nevertheless, the performance’s stability requires further examination. A double-network hydrogel coating, characterized by a double-network structure, consolidates the properties of two types of hydrogel, thereby enhancing its antifouling and mechanical properties. This makes it particularly suited for maritime antifouling applications. However, there is a need to bolster the design to widen its applicability and reliability. The amphoteric ionic hydrogel coating, due to its bipolar ionic properties and balanced surface charge, exhibits remarkable anti-bio-adhesion and anti-protein adsorption. The current studies focus on exploring combinations of different ionic properties to optimize performance and stability.

This review commences with an overview of the marine antifouling background, followed by a detailed introduction to the structures, qualities, and categorizations of hydrogels. It proceeds to encapsulate the progress in improved hydrogel antifouling coatings research in recent years and the influence these advancements have had on prospective research orientations. In conclusion, given the significant research value of hydrogels in the antifouling domain, this review examines their structural properties and the current research status of hydrogel materials, while summarizing the methods that enhance their antifouling performance. It also ushers in a major breakthrough in the marine biofouling field.

## 2. Basic Overview of Hydrogels

### 2.1. Structure of Hydrogels

**Figure 1:**
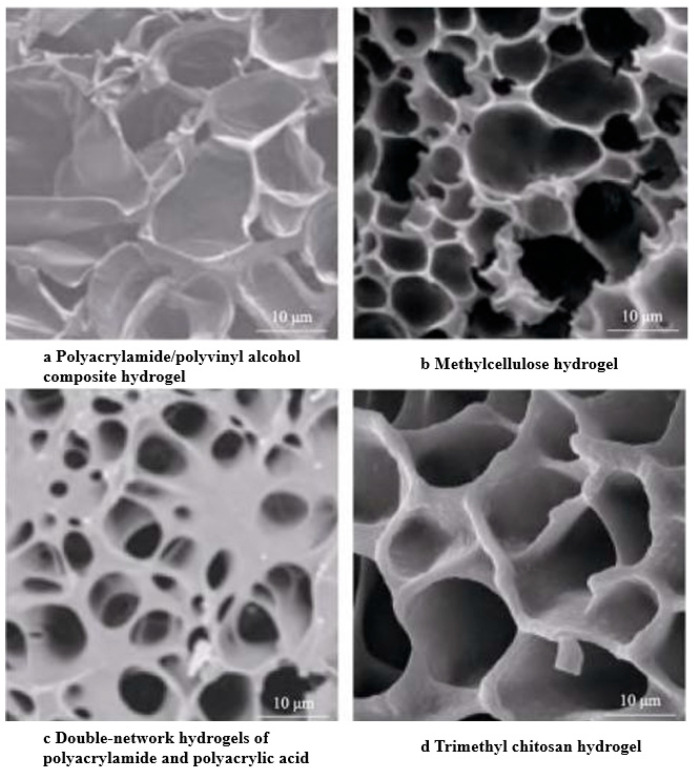
Photographs of hydrogel scanned by electron microscope should be listed as: (**a**) Cross-sectional micromorphology of polyacrylamide/polyvinyl alcohol. (**b**) Cross-sectional micromorphology of methylcellulose hydrogels. (**c**) Cross-sectional micromorphology of polyacrylamide and poly(acrylic) bi-network hydrogels. (**d**) Cross-sectional micromorphology of trimethyl chitosan hydrogels [19,20,21,22].

Hydrogels exhibit a three-dimensional network-like structure, as depicted in Figure 1. The cross-sectional microscopic morphology of composite hydrogels, such as polyacrylamide/poly(vinyl alcohol) [19], methylcellulose hydrogels [20], bi-networked hydrogels of polyacrylamide and poly(acrylic acid) [21], and trimethyl chitosan hydrogels [22], reveals the hydrogels’ porous texture, underlined by an intrinsic three-dimensional network-like design. This intricate three-dimensional spatial organization allows for incorporating corrosion inhibitors, drugs, and electrolytes, thereby broadening their potential usage in corrosion protection and surface engineering. Different hydrogels have different structural characteristics.

#### 2.1.1. Pore Structure Characteristics of Macroporous Hydrogels

A macroporous hydrogel is a hydrogel structure with pore diameters exceeding 10 um [23]. Regarding the morphology of pores, hydrogel scaffolds prepared by different methods will present different pore morphologies: for example, 3D printing technology can prepare pore morphologies, such as rectangular, circular, hexagonal, etc.; the pore morphologies prepared by lyophilization and foaming methods are more irregular; and the pores obtained via the pore maker method are presented in the form of a pore maker. Regardless of the pore morphology of hydrogel scaffolds, their surface curvature is one of the important factors affecting the tissue morphology and growth rate. Within a certain range, higher curvature of the inner surface of the scaffold leads to faster growth of tissue cells. In addition, a more regular arrangement of pores also contributes to the repair effect of the tissue.

#### 2.1.2. Structural Characteristics of PEAS-PEG Microgels

**Figure 2:**
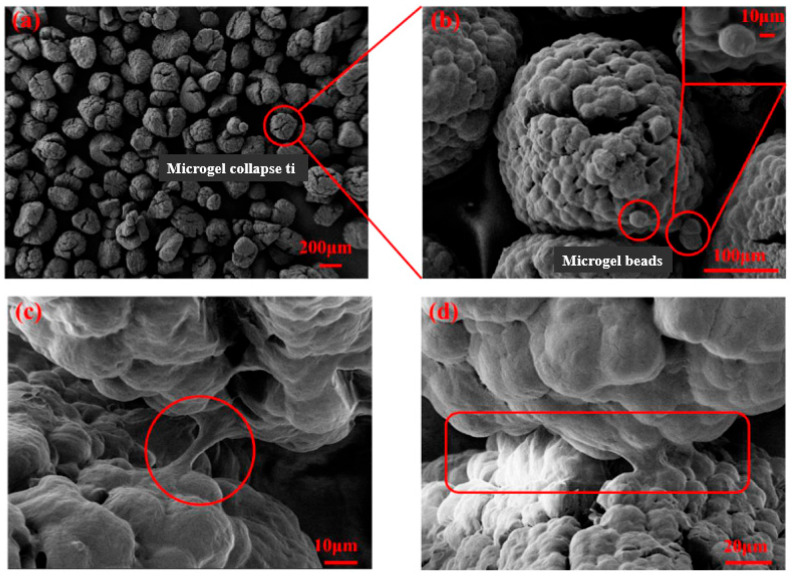
SEM morphology of PEAS-PEG microgel should be listed as: (**a**) There are many evenly dispersed particles, and these particles are formed by the collapse of many microgel globules. (**b**) Cracks can be seen in the middle of the microgel slump, which is in the process of vacuum freeze-drying. (**c**) There are cross-linking points between the microgel slumps. (**d**) Cross-linking points can be seen between the microgel slumps [24].

The SEM (scanning electron microscope) surface morphology of PEAS-PEG microgels (Poly St@ 2-Hydroxyethyl methacrylate(HEMA)-POLY(ETHYLENE GLYCOL)(N) MONOMETHACRYL ATE(PEGMA) microspheres with St as the core and HEMA and PEGMA as soap-free emulsion polymerizationm, called PEAS microspheres prepared the shell), as shown in Figure 2, was obtained by Zhang et al. [24]. In Figure 2a, it can be seen that there are many uniformly dispersed granules, which, in turn, are formed by the collapse of many microgel pellets. As shown in Figure 2b, cracks can be seen in the middle of the microgel slumps, which are caused by the removal of solvent from the PEAS-PEG microgel polymer solution during vacuum freeze-drying; each microgel slump has a particle size of about 290 μm, and each microgel pellet forming a slumped sphere has a particle size of about 26 μm. Free microgel pellets aggregated by bridging generate a certain osmotic pressure [25], and the enrichment of microgel pellets tends to stabilize when the microgel concentration is high enough.

#### 2.1.3. Structural Characteristics of PVA/(Polyacrylamide)PAAm Hydrogel

**Figure 3:**
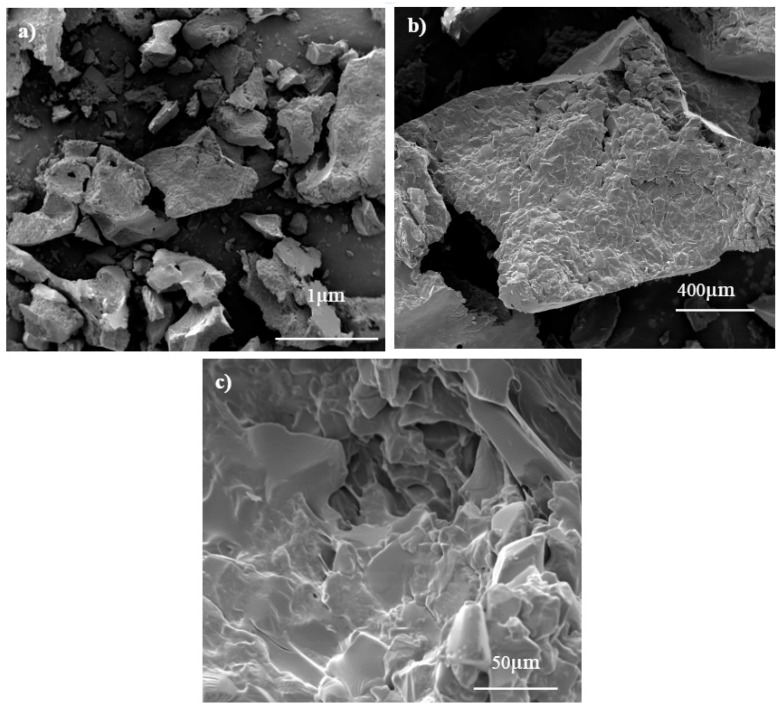
SEM images of dry PVA/PVVm hydrogel particles should be listed as: (**a**) Hydrogel particles after water absorption equilibrium. (**b**) The external profile of the hydrogel particles after water absorption equilibrium. (**c**) Partial enlargement [26].

After the PVA/PAAm hydrogel particles are embedded in the epoxy resin matrix, seawater slowly penetrates the coating from tiny pinholes on the surface of the epoxy resin, and the PVA/PAAm hydrogel particles swell in contact with water, forming a low-surface-energy, super-lubricating hydrogel adhesive water film layer on the surface of the coating, and the uniform water-absorbing array formed by the composite of the PVA/PAAm hydrogel micro-powder and resin is the basis of the preparation of the array hydrogel-modified coatings [26]. The formation of uniform water-absorbing arrays of PVA/PAAm hydrogel micro-powder composite with resin is the basis for the preparation of the array-type hydrogel-modified coating [24]. Figure 3 [26] shows the SEM photographs of dry PVA/PAAm hydrogel micro-powders. From Figure 3a, it can be seen that the hydrogel micro-powder shows irregular particle shapes and flakes, and the edges of the hydrogel particles are not smooth and rough due to the large impact of mechanical crushing. Figure 3b is an SEM photo of hydrogel particle morphology with the local magnification of 100-times. It can be seen that the surface of PVA/PAAm hydrogel micro-powder carries more angles, which can make the surface area increase, and the observation of single particle magnification of 1000-times reveals that the surface of the particle presents a rough and uneven state (Figure 3c). From the microscopic morphology observation of PVA/PAAm hydrogel particles, it can be seen that the surface of PVA/PAAm hydrogel particles is irregularly shaped with angles and a rough surface, which increases the bonding area between PVA/PAAm hydrogel particles and the base epoxy resin, improves the bonding force between PVA/PAAm particles and the film-forming resin, and facilitates the bonding force of PVA/PAAm hydrogel particles in the dynamic seawater. The PVA/PAAm hydrogel particles can be retained on the surface of the coating for a long time without being extruded under the action of complex external forces. After the coating absorbs water, it maintains a complete and continuous solidified water film layer.

A hydrogel is a water-swelled entity featuring a reticulated structure. Upon water absorption, it exhibits a degree of elasticity and remains undissolved in water [27]. Research has shown that the unique attributes of hydrogels, such as water absorption, water retention, non-toxicity, eco-friendliness, and the ability to become smooth and easily peelable post-water-absorption, are akin to the mucus layer secreted by large marine organisms [28]. Antifouling coatings, crafted from hydrogel-based materials and applied to the surfaces of ships and other marine structures, mimic the epidermal mucous membranes of larger marine organisms post-water absorption, so this similarity aids in the prevention of adherence by marine organisms in aquatic environments [29]. Consequently, hydrogels constitute an ideal choice for marine antifouling coating materials [30].

### 2.2. Properties of Hydrogels

#### 2.2.1. Basic Physical and Chemical Properties

Hydrogel materials, characterized by their unique water-filled cross-linking network structure, possess an array of distinct properties. The hydrophilicity of these hydrogels, attributed to the preponderance of hydrophilic groups within their skeletal polymers, manifests in a significant contact angle that renders oil adherence challenging, thus presenting the potential for the advancement of marine antifouling coatings. Simultaneously, the hydrogel’s inherent non-enclosed internal structure facilitates osmosis, enabling the formulation of oil–water separation coatings. In addition, hydrogels boast noteworthy properties, like low friction, light transmission, and biocompatibility. Given the escalating concern for marine environments, the conception and study of environmentally friendly novel antifouling coatings have emerged as a principal trend with hydrogels, due to their soft, hydrophilic quality, garnering increased attention [31].

1. Water Absorption

Hydrogels are highly hydrophilic and can form a hydrated layer to inhibit biofouling. The water it stores consists of three components: bound water adsorbed by hydrophilic functional groups, such as hydroxyl, carboxyl, amino, carboxylic ester, and acyl groups on the polymer molecular chain; water molecules adsorbed directly on the hydration sites called intermediate water; and free water that enters into the structure of the hydrogel network driven by the osmotic pressure [32,33] (Figure 3a). The amount of bound water is dependent on the number of functional groups and the rate of bonding with water molecules.

**Figure 4:**
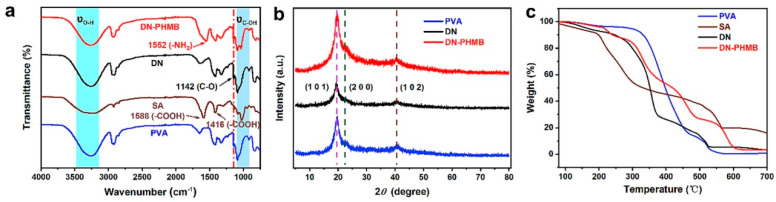
Characterization and morphology of hydrogel, should be listed as: (**a**) FT-IR spectra of prepared hydrogels. (**b**) XRD spectra. (**c**) TGA curve [32].

Zhang et al. [32] found that the oxygen atom of water in the polymer network can absorb six water molecules, while the amino or hydroxyl group in the molecular chain can only absorb one water molecule through hydrogen bonding, due to the ligand bonding formed by Al^3+^ (Figure 4b). Therefore, the amount of bound water adsorbed by Al^3+^ coordinated chitosan (CS-Al^3+^) hydrogels is higher compared to pure chitosan hydrogels.

The O-H absorption peak of the hydrogel was about 3200 cm^−1^ and the C-O absorption peak was about 1085 cm^−1^ (Figure 4a). Compared with the pure PVA hydrogel, no new absorption peaks were observed in the DN hydrogel, which indicated that the reaction was purely physical, without any chemical changes. The tensile vibrational absorption peak-1 at 1142 cm for C-O in the microcrystalline phase region of the PVA hydrogel is a characteristic absorption peak common to the PVA, double network (DN), and double-network Polyhexamethylene Biguanide Hydrogels (DN-PHMB) [34,35], the intensity of which is directly associated with the X-Ray Diffraction (XRD) spectra of PVA, DN and DN-PHMB hydrogels, highlighting three diffraction peaks, with two θ peaks appearing at 19.6°, 22.9° and 40.8°, indicating the (101), (200) and (102) crystal planes of the PVA microcrystals (Figure 4b) and confirming the production of microcrystalline regions of the PVA network via drying and re-solubilisation. The PVA in DN hydrogels is a cross-linked network formed through microcrystalline regions. In addition, the diffraction peaks of PVA microcrystals in DN and DN-PHMB hydrogels are identical to those of pure PVA hydrogels, suggesting that the structure of DN does not affect the formation of crystalline regions in PVA hydrogels. Thermogravimetric analysis (TGA) of PVA, SA, DN, and DN-PHMB hydrogels, as shown in Figure 4c, suggests that the first plateau of weight loss between 80 and 150 °C is likely to be attributed to the loss of bound water in the hydrogels. The first plateau of weight loss between 80 and 150 °C may be attributed to the loss of bound water in the hydrogels. All hydrogels exhibit a second plateau of weight loss starting at 200 °C.

2. Antimicrobial properties

Hydrogels have been extensively studied in wound dressings and antimicrobial agents to prevent wound infection and promote wound healing by introducing bactericidal substances and cellular actives [36,37]. Shi et al. [2] prepared a fully physically crosslinked double-network hydrogel of sodium alginate/chitosan/zinc ions (SA/CS/Zn)^2+^ Pyruvate Dehydrogenase(PDH), free of any toxic chemical reagents. The mechanical properties of PDH were significantly improved compared to sodium alginate/chitosan single-network hydrogels cross-linked by electrostatic interactions. It was found that with an increasing zinc content, the rate of gelation increased, the rate of swelling decreased, and the mechanical properties improved. PDH showed good antibacterial activity against both Staphylococcus aureus and Escherichia coli. Patel et al. [37] synthesized a spherical cellulose nanocrystalline (s-NC)-enhanced carboxymethyl chitosan-based injectable and adhesive hydrogel for rapid skin rejuvenation. S-NC exhibited better cellular activity than the cellulose nanocrystals for better cellular activity. The skin regeneration potential of the hydrogel scaffolds was also examined in rats using a wound healing model. The composite scaffolds also showed better antimicrobial potential. Stable and suitable phospholipid vesicles containing quercetin and curcumin, i.e., liposomes and vesicles containing permeation enhancers (PEVs), were prepared by Castangia et al. [38]. In vitro and in vivo tests highlighted the excellent effect of quercetin and curcumin nanovesicles in counteracting the lesions and inflammation induced by the phorbol ester 12-O-tetradecanoylphorbol-13-acetate (TPA). Myeloperoxidase activity, which is used to measure inflammation, was significantly inhibited by quercetin liposomes (59%) and curcumin liposomes as well as PEG-PEV (~68%). Hydrogels can exhibit good antimicrobial properties in the medical field. In marine antifouling, good antimicrobial properties can reduce the adhesion of microorganisms on ship surfaces. The antimicrobial properties of hydrogel in medicine can promote the application of hydrogel in marine antifouling.

3. Mechanical properties

Hydrogel has a large water content and weak intermolecular cross-linking, so it is important to improve the mechanical properties to extend its application. The borate ester bond has a strong steric coupling in the structure, which is conducive to the improvement in hydrogel mechanical properties. Chen et al. [39] designed a bi-dynamic chemically cross-linked hydrogel based on acyl hydrazone and borate ester bonds, which utilizes the interactions of PVA with boric acid ions and acyl hypoxia bonds to form a stable and tough hydrogel. The hydrogel has good self-healing properties and high mechanical strength, which can be used for repulsive tissues, sensors, and other smart materials. Cross-linking cellulose nanofibers (CNFs) based on borate ester bonding can effectively improve the mechanical properties of cellulose-based hydrogels. The CNF has good mechanical strength, and its abundant hydroxyl groups are conducive to chemical modification and composite material preparation, which can endow the hydrogel with good mechanical properties [40]. Zhong et al. [41] prepared modified carboxymethyl cellulose (OCMC-DA) hydrogels synergistically reinforced with CNF using PVA-borax gel as a matrix, and dynamically reversible borate and hydrogen bonds were formed between OCMC-DA, PVA, and CNF, which resulted in a much stronger hydrogel.

**Figure 5:**
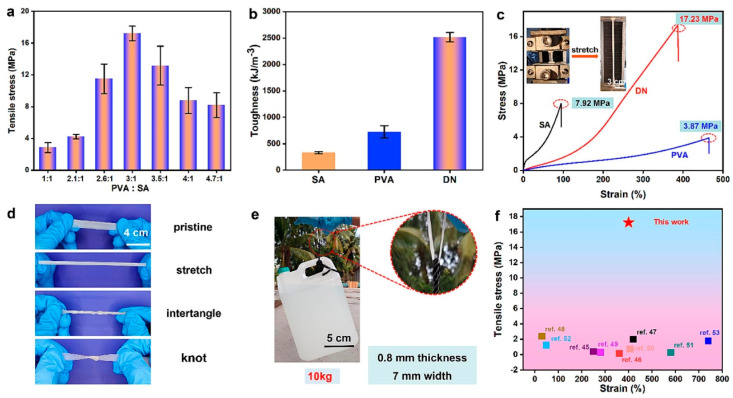
Mechanical properties of hydrogels should be listed as: (**a**) Tensile strength of hydrogels with different PVA/SA ratios. (**b**) Toughness of prepared hydrogels. (**c**) Strain–stress curve. (**d**) Digital photograph of DN-PHMB hydrogel tensile measurements. (**e**) Image showing that DN-PHMB hydrogel can lift a heavy object. (**f**) Comparison of the mechanical properties of DN-PHMB hydrogel with those of reported hydrogels [17,45,46,47,48,49,50,51,52,53].

The mechanical properties of the hydrogels prepared in this study were amenable to uniaxial tensile tests (Figure 5) [17]. Figure 5a shows that the tensile strength of DN hydrogels increases and then decreases with increasing PVA content. The maximum tensile strength of DN hydrogel was 17.23 MPa, and the elongation at break was 388.35% when the ratio of PVA to SA was 3:1. The PVA network endowed the DN hydrogel structure with a good strain capacity, and the SA network enhanced the mechanical strength of the overall structure. During deformation under tensile stress, the two physical cross-linking networks in the DN structure can act as energy dissipation networks through energy transfer, thus preventing external damage (Figure 5b). As can be seen in Figure 5c, the tensile strengths of the PVA and SA single-network hydrogels were 4.12 MPa and 7.92 MPa, and the elongation at break was 464.59% and 95.00%, respectively. However, the mechanical strength of DN hydrogel is 17.23 MPa, which indicates that it is not a simple ‘1 + 1 = 2’ linear superposition of the mechanical strengths of the two single-network hydrogels but a nonlinear superposition of the ‘1 + 1 > 2’ binary structure. The water contents of PVA mono-network hydrogel, SA mono-network hydrogel, and DN hydrogel were 63.39%, 54.99%, and 58.04%, respectively. Figure 5d shows that DN hydrogels can be easily transformed into various shapes (stretching, tangling, knotting), without causing damage, which verifies the strong mechanical properties of DN hydrogels from different perspectives. Figure 5e shows that the DN hydrogel can withstand up to 10 kg of weight, which further confirms the high strength of the hydrogel. DN-PHMB hydrogels showed good mechanical properties (Figure 5f) compared to the common dual-network hydrogels reported so far [42,43,44,45,46,47,48,49,50], suggesting that DN-PHMB hydrogels have a promising future in various application scenarios.

Although many of the properties of hydrogels are described above, their development across various domains has been hindered due to the deficient mechanical properties they possess. Enhancing the mechanical features of hydrogels, without compromising their other good properties, is presently a crucial area of research within the hydrogel field. High-toughness composites are achievable through interfacial bonding, and, similarly, there is potential for noteworthy enhancements in fiber materials’ fatigue resistance too [51].

#### 2.2.2. Special Property

The structural properties of hydrogels give them unique properties. For example, most hydrogels exhibit a relatively rapid ability to heal themselves after injury, a property that makes them suitable for the manufacture of “mechanical skin” [52]. In addition, hydrogels have the potential to be applied in the field of marine antifouling coatings due to their commendable irritation response properties and injectable properties [53]. When exposed to specific stimuli, hydrogels are designed to have an appropriate response, expanding their range of applications in areas such as drug release control and corrosion protection [53]. Due to the self-healing properties, stimulus-response properties, and injectability of hydrogels, they can be applied to the marine antifouling of ship-made objects, which provides great potential for marine antifouling of hydrogels [53].

1. Self-healing properties

**Figure 6:**
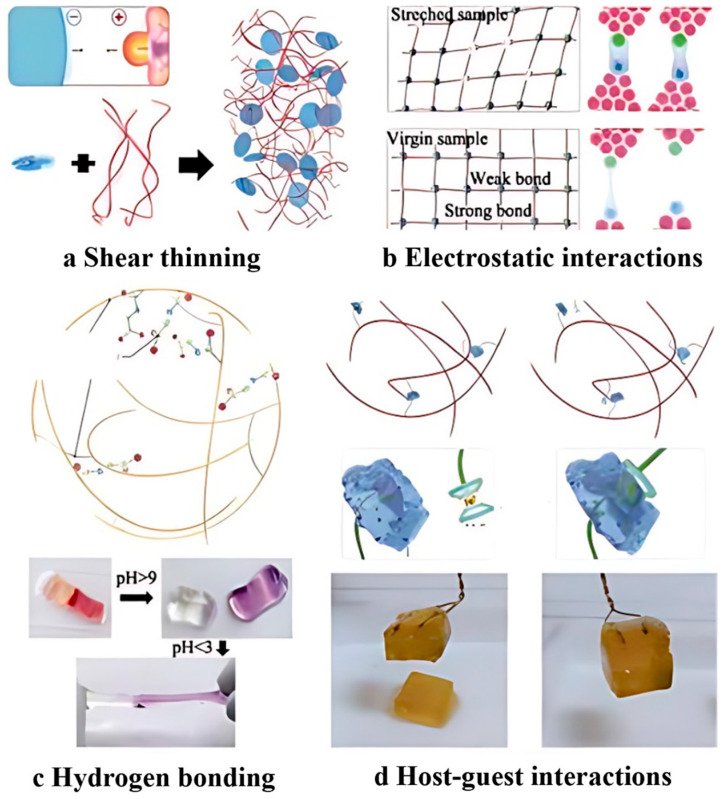
Self-repair diagram of polymerized mesh structure in hydrogel (**a**) Shear-thinning hydrogel through nanocomposite. (**b**) Ionic interactions. (**c**) Hydrogen bonds. (**d**) Host–guest coupling [53].

The self-healing capability stands as a distinctive attribute of hydrogels [52]. Due to their exceptional self-repair properties, energetic hydrogels find potential applications in surface treatments, mechanical skins, and antifouling coatings [53]. On encountering damage, the intricate polymeric meshwork within the hydrogels can initiate a self-repair process, leveraging mechanisms, such as shear thinning, electrostatic interactions, hydrogen bonding, and host–guest interactions, illustrated in Figure 6 [53]. Self-repairing hydrogels have a cross-linked polymer network structure, which can autonomously repair from damage, which is conducive to prolonging the life of the material and restoring or preserving its structural original properties, showing great potential for application in the fields of coatings, drug delivery carriers, bionic skins, and soft robots [54].

B(0H) and polyhydroxy polymers (e.g., PVA, cellulose) can form dynamically reversible borate ester bonds, resulting in the preparation of hydrogels with self-healing properties. Chen et al. [55] used a one-step method to prepare borate ester-bonded cross-linked hydrogels. It was found that dynamic reversible borate ester bonds were formed between boric acid and hydroxyl groups under alkaline conditions, and the hydrogels were able to achieve complete self-healing 48 h after damage, whereas the self-healing ability of the hydrogels was weaker under acidic conditions. Cheng et al. [56] constructed a dual-network cross-linked hydrogel by the borate ester bonds formed between PVA and borax and the acylhydrazone bonds formed between aldehyde-based nano fibrillar cellulose and modified sodium alginate, with 60 min fast self-healing properties. Ji et al. [57] prepared hydrogels with strong mechanical properties, aiming at self-healing properties by copolymerizing dimethylaminoethyl methacrylate (DMAEMA) with N-hydroxymethacrylamide (NAM) as an alkaline monomer, with the assistance of benzene di-boronic acid (BDBA). Mechanistic studies revealed that under alkaline conditions, the free hydroxyl groups of the borate bonding network structure in the copolymer and B(OH) can be exchanged by free hydrolysis and re-esterification of the bonds, thus conferring self-healing ability to the hydrogel, which was able to recover the strain and strength properties after fracture for 100 h. Ghosh et al. [58] utilized guanosine (G) with naphthalene boronic acid, which was synthesized under the induced synthesis of the monovalent cation, K*, via multicomponent self-assembly and borate bonding to form G-quaternary-based hydrogels and confirmed the self-healing property of hydrogels related to the rapid transition between its in situ cyclic borate bond formation and hydrolysis.

Several scholars have crafted hydrogels featuring a three-dimensional network structure leveraging silver-mercapto coordination, thereby conferring good self-repairing qualities to the gel material [59]. Such a material shows promise for application as self-repairing coatings. Notably, these kinds of hydrogel materials also possess antimicrobial properties [60] and could be employed as antimicrobial coatings. Beyond silver-mercapto coordination, hydrogen bonding is another frequently utilized mechanism by scholars worldwide for formulating hydrogel materials that encapsulate self-healing properties. Rong et al. [61] reported the preparation of an antifreeze conductive hydrogel system with self-healing facets via hydrogen bonding. Zhao et al. [62] harnessed the self-assembly of SA within a polyacrylamide (PAM) network, contributing to the creation of a PAMSA hydrogel with a semi-interpenetrating network structure through hydrogen bonding. In a similar vein, In et al. [63] employed the Schiff base reaction of an amino group on hydroxy butyl chitosan (HBC) and the aldehyde group on oxidized konjac glucomannan (OKGM) to fabricate self-healing hydrogels. Conventional cross-linking structures primarily comprise single covalent bonds, lacking the capability for self-repair post-damage. To acquire this self-repairing attribute, the cross-linking structure ought to be dynamically reversible and relatively facile to open up, rendering the attainment of a robust and highly dynamic reversible cross-linking structure as a current research focal point [64].

2. Stimulus Response Characterization

Intelligent stimulus-responsive hydrogels exhibit outstanding sensitivity to subtle alterations in the external setting, undergoing relevant modifications in their physical configuration or chemical characteristics [65]. This category encompasses temperature-responsive, light-responsive, magnetic-responsive, and pH-responsive hydrogels [65]. Owing to its extensive application potential, temperature-sensitive hydrogel has recently emerged as the prominent focus in environment-responsive hydrogel research [66]. Certain polymer solutions respond to temperature fluctuations via a reversible sol–gel transition, an intrinsic trait facilitating the controlled release of encapsulated corrosion inhibitors [67].

**Figure 7:**
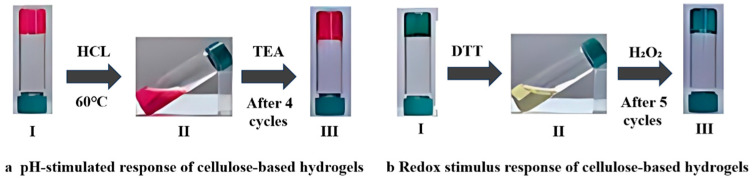
Schematic representation of cellulose-based hydrogel response [69].

Cui et al. [68] undertook a holistic exploration of the gel configuration and mechanism of thermosensitive hydrogel systems utilizing both simulation and experimental methodologies. Yang et al. [69] crafted a cellulose-based hydrogel exhibiting dual responsiveness towards pH and redox stimuli. As Figure 7 illustrates, the sol–gel transition in pH-simulated gels occurs when the red-stained hydrogel morphs into a solution upon the addition of a hydrochloric acid solution, subsequently returning to a gel state upon triethanolamine (TEA) incorporation [69]. Figure 5b delineates the sol–gel transition for redox-simulated gels: the methylene blue-stained hydrogel reverts to a solution state following the introduction of dithiothreitol (DTT), reverting to a gel upon the subsequent addition of hydrogen peroxide (H_2_O_2_). Moreover, this cellulose-based hydrogel is capable of leveraging the reversibility of acyl hydrazone and disulfide bonds to effectuate the sol–gel transition. The hydrogel may also serve as a carrier for corrosion inhibitors, thereby proposing novel applications in corrosion protection. Di et al. [70] devised a composite hydrogel with shape memory features through chemical–physical cross-linking, using photothermal agents such as polydopamine (PDA), carboxymethyl cellulose (CMC), and PVA. Taking advantage of the photothermal conversion effect of PDA nanoparticles, near-infrared radiant energy was converted into thermal energy, which triggered a temperature increase, eventually leading to hydrogen bond dissociation and shape restoration of the hydrogel. This highlights its profound implications for environmentally friendly materials pertinent to antifouling coatings for vessels.

Liu et al. [71] synthesized pH-responsive composite hydrogels by employing pH-responsive reversible catechol-boronic acid bonds established between chlorinated catechol and phenylboronic acid. Under acidic environments, the catechol-boronic acid bond undergoes dissociation, imbuing the hydrogel with bactericidal properties. In contrast, under alkaline conditions, the formation of catechol-borate bonds occurs, which exhibits no cytotoxicity. The bactericidal capabilities of these hydrogels are, thus, modifiable through pH stimuli. Furthermore, supramolecular hydrogels demonstrate notable pH responsiveness [72], and the nanostructures formed therein can be manipulated by varying the concentration of the monomer. This leads to the formation of a spectrum of structures, from helical nanofibers to other types of pH-responsive hydrogels [73].

Stimulus-responsive hydrogels display marked differences in their swelling behavior in response to diverse environmental conditions. This prominent quality can function as a control switch or be exploited for loading corrosion inhibitors for robust metal corrosion protection. This capability offers durable defense for metals and safeguards the coatings while the ship navigates through marine environments, consequently mitigating the consequences of biofouling on oceanic ecosystems.

3. Injectability

Injectable hydrogels have great potential for development in drug delivery and wound dressings. The good injectability of hydrogels not only allows them to be more evenly distributed in vitro but also allows for better control of target sites in vivo. The development of environmentally stable hydrogels is of great significance, as harsh conditions such as humidity, underwater environment, and high temperatures can reduce the performance of hydrogels and, therefore, have potential for marine antifouling.

Borate bonds can be quickly re-bonded in situ after disruption, a property that confers good injectability to borate-bonded cross-linked hydrogels. Du et al. [74] prepared a bi-network hydrogel with injectability in humid environments by using aminophenylboronic acid-modified oxidized sodium alginate (OSA-BA), PVA, and dopamine (DA). The dynamic phenylboronate bond formed between the PVA and OSA-BA bond formed by PVA and OSA-BA, and the Schiff base network formed by OSA-BA and DA could confer effective self-healing properties to the hydrogel. Due to the wet adhesion property of DA and the underwater reconstruction property of borate bonds, the hydrogels exhibited rapid in situ reformability after being injected into different environments. Borate ester-based hydrogels constructed from bio-based materials have good biocompatibility and can be used as injectable biomaterials. Ding et al. [75] prepared a collagen-based multidynamic network hydrogel using collagen, bis-formaldehyde guar gum, and borax, with dynamic imine bonding between the bis-formaldehyde guar gum and collagen, dynamic diol borate ester bonding between borate ions and the hydroxyl groups of the guar gum, and collagen-guar gum non-covalent cross-linking (hydrogen bonding and entanglement) coexisted in the hydrogel network, which endowed the hydrogel with good injectability and rapid self-repair capability. Meanwhile, collagen-based hydrogels have the property of accelerating wound healing and can be applied as wound dressings. Huang et al. [76] prepared GG@PANI(Fe)-borax hydrogels cross-linked by borate/diol bonds by cross-linking guar gum (GG) with iron-ion doped polyaniline (PANI(Fe)), which was then simply mixed with borax. The hydrogel exhibited shear-induced flow and transient recovery due to the presence of borate bonds, and this shear-thinning behavior conferred injectability to the hydrogel. In addition, the bioactive iron ions contained in the hydrogel can promote fibroblast proliferation and revascularisation, thereby accelerating tumor wound healing.

### 2.3. Classification of Hydrogels

The hydrogels are classified according to their source, as shown in Table 1.

#### 2.3.1. Natural Source Hydrogel

Proteins, oils, and polysaccharides serve as the primary biopolymers deployed in the formulation of hydrogels, utilizing natural polymers or biopolymers derived from plant and animal bodies as the scaffold for synthesizing high-grade hydrogels [80]. Due to their plentiful abundance, exceptional biocompatibility, and commendable degradability, natural polysaccharides and proteins are deemed ideal constituents in hydrogel preparation [81,82]. Notably, polysaccharide-based hydrogels exhibit favorable electrical conductivity, antimicrobial function, shape memory features, self-healing capabilities, outstanding water retention attributes, thermal conductivity, and thermal stability [83], thus garnering considerable interest in marine antifouling applications.

Regarding marine antifouling applications, polyvinyl alcohol-poly(acrylic) (PVA-PAHX) hydrogel represents a practical application of hydrogel coatings, demonstrating high mechanical strength, a reduced dissolution rate, and antifouling effects, as stipulated by the hexamethylene bisacetamide (HMBA) content in the dual-network (DN) hydrogel [77]. Li et al. [77] established that DN hydrogels could be produced using HMBA (a derivative of capsaicin N-(4-hydroxy-3-methoxybenzyl) acrylamide) within a polymer matrix. A propensity for good hydrophilic properties is vital for polyvinyl alcohol (PVA) to function as a pliable network, while the rigid network is constructed by the polymerization of acrylamide (AM) and HMBA.

#### 2.3.2. Synthetic Hydrogel

Hydrogels are fabricated via chemical methods such as polymerization and grafting, tailored with distinctive features to align with diverse application needs [24]. Synthetic hydrogels, engineered by modifying the structure of natural polymer monomers, are prevalently employed in marine antifouling due to their enhanced mechanical strength, commendable chemical stability, and robust mechanical characteristics [78]. Despite their stability, synthetic hydrogels suffer from a lack of cell recognition features and inferior biocompatibility, thereby broadening the application scope for natural hydrogels [84].

Under the examination of Cong et al. [85], macroporous hydrogels exhibited effective marine antifouling properties after 5 months and maintained their mechanical strength. The team fabricated PVA/PAM macroporous hydrogels from polyvinyl alcohol, PEG6000, PEG1000, and acrylamide (AM), leveraging a pore-forming technique. In a different study, Pontus et al. [86] inspected the application of light-cured thiolene hydrogel coatings drawn from polyethylene (methyl glycol) (PEG) for marine antifouling purposes. Katsu Yama et al. [87] demonstrated that the germination of algal swimming spores could be impeded by polyelectrolyte hydrogels. Rasmussen et al. [88] presented findings on the antifouling influences of certain natural polymer gels and chemically cross-linked polyvinyl alcohol (PVA) gels on barnacle carp.

#### 2.3.3. Hybrid Hydrogel

Hybrid hydrogels, serving as composite polymers, harness the attributes of both natural and synthetic hydrogels. The optimization of structural and molecular organizations boosts physical, electrical, chemical, and biological facets [79,89,90]. Owing to the presence of polymer materials, inorganic materials, and diverse polymer materials of sub-1 μm scale, the components mesh thoroughly, enabling the hybrid hydrogel to preserve a distinctive three-dimensional structure. As a result, they are also categorized as water-swellable materials, offsetting the limitations of traditional hydrogels [79]. Recent research has seen widespread usage of hybrid hydrogels in marine antifouling applications, owing to their enhanced mechanical performances, commendable tolerance, water retention, cost-effective nature, and their role as effective adsorbents [79]. A study revealed that a freshly devised anionic adsorbent, when partially alkaline hydrolyzed by 0.5 M NaOH, escalates nitrate adsorption on being laden with Fe^2+^. This cationic and anionic adsorbent was concocted by Chauhan [91] et al. in lignocellulose for marine antifouling. The team employed acrylamide and N, N-methylenebisacrylamide along with pine needles and their carboxymethylated versions to establish network hydrogels.

## 3. Advances in Modified Hydrogel Coatings

Hydrogel research has surged in recent years, invigorating the field of marine antifouling. Numerous investigations aim to mitigate marine pollution by creating innovative antifouling materials, thereby tailoring hydrogel structure and attributes. Hydrogel coatings have shown potential for application in various fields due to their good moisturizing properties, biocompatibility, and adaptability. However, effectively applying hydrogel coatings to various material surfaces and maintaining their performance present some challenges. Firstly, the diverse physical and chemical properties of hydrogels make it complex to optimize their lubricating performance; secondly, the adhesion stability of hydrogels to different substrates is a technical challenge, as the structure and formation methods of hydrogels vary; moreover, improving the durability, antifouling ability, and mechanical strength of hydrogel coatings needs to be addressed urgently. To meet these challenges, scientists are exploring a variety of modification methods. Among them, research on enhanced hydrogel coatings has become a focus of attention. Key modifications have yielded five principal coating types: PVA-based hydrogel coatings, PEG-based hydrogel coatings, hydrogel-organic resin hybrid network coatings, dual-network hydrogel coatings, and amphiphilic ionic hydrogel coatings. By boosting cross-linking, PVA-based hydrogel coatings amplify mechanical resilience; PEG-based hydrogel coatings devise hybridized network structures to counterbalance their mild durability shortfall compared to other coatings; hydrogel-organic resin hybrid network coating elevates performance steadiness, guaranteeing long-term usage; the lure of dual-network hydrogel coatings lies in their broadened application scope; amphoteric ionic hydrogel coatings exploit varying ion properties to fine-tune the coating’s performance and stability. Therefore, myriad modifications to hydrogels unfold new avenues for marine antifouling.

### 3.1. PVA-Based Hydrogel Coatings

PVA-based materials, characterized by toughness, inherent film-forming ability, and formidable mechanical strength, are implemented extensively across the domains of separation and purification, packaging, biomedicine [92], and marine antifouling [93]. PVA hydrogel, originating from PVA aqueous solution, forms conservatively under natural circumstances. The weak water resistance and mediocre mechanical properties, which are common issues amongst naturally formed hydrogels, inhibit the application of such hydrogels in marine antifouling endeavors. Consequently, physical cross-linking [94] or chemical cross-linking [95] prove necessary. In instances where hydrogel coatings are sought for metals, addressing the hydrogel’s adherence capability also presents a significant concern.

**Figure 8:**
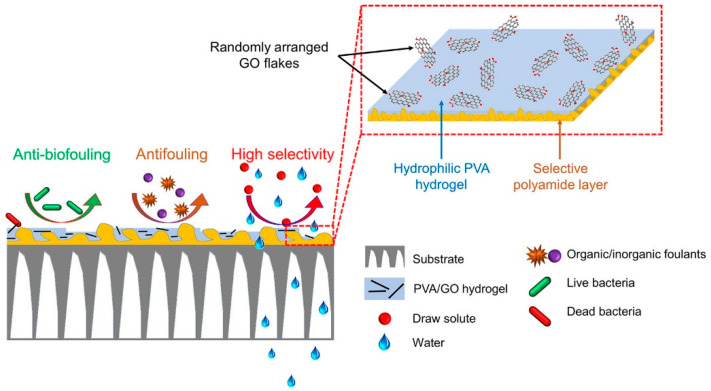
PVA/GO coating [97].

Xu et al. [96] fabricated PVA hydrogel coatings via chemical cross-linking, employing water, starch, and polytetrahydrofuran dibenzoate as primary materials, with citric acid acting as the cross-linking agent and a melting method being utilized for the plasticization of PVA. The experimental results demonstrated that once the mass fraction of citric acid reached 5%, there was a marked increase in the cross-linking density. Alongside this increase, the stability of the cross-linking structure improved, and the mechanical properties of the PVA hydrogel coatings were considerably enhanced. Furthermore, as depicted in Figure 8, Akther et al. [97] synthesized a hydrophilic composite hydrogel utilizing graphene oxide (GO) and glutaraldehyde cross-linked PVA. Subsequently, they selected the polyamide layer of a widely used permeable membrane and layered it with the composite hydrogel. The characteristics, functionality, and antibacterial attributes of the forward osmosis membrane can be fine-tuned by modifying the GO concentration in the PVA hydrogel coating. Hydrogel-embedded forward osmosis membranes exhibit solute retention, decontamination efficacy, and antimicrobial traits, with a notable 82% decrease in bacterial adherence when compared to untouched membranes. In a related study, Wang et al. [98] engineered a poly(vinyl alcohol)/poly(acrylamide) (PVA/PAAM) hydrogel micro-powder via physical cross-linking. This was subsequently combined with an epoxy resin to produce an antifouling coating enhanced with PVA/PAAM hydrogel micro-powder. Upon the formation of the coating, PVA/PAAM hydrogel micro-powder develops a compact array of water-absorbing nodes on its surface, exhibiting its inherent bacteriostatic, superoleophobic, and lubricating traits, with the ideal quantity of PVA/PAAM hydrogel micro-powder to be incorporated being 10%. In the anti-microalgae adhesion trial, the microalgae cell adhesion density for the epoxy resin coating stood at 3.2 × 10^4^ cm^−2^, in stark contrast to less than 0.1 × 10^4^ cm^−2^ for the epoxy resin coating integrated with hydrogel micro-powder. Concurrently, the PVA/PAAM hydrogel micro-powder can act as a vehicle for the fungicide Cu_2_O, facilitating control of Cu_2_O discharge through the porous structure of the hydrogel, thereby granting the coating extended antifouling longevity. The trio of studies utilized cross-linking methods to refine the natural hydrogel, culminating in attributes, robust mechanical properties, and antimicrobial qualities benefiting marine antifouling, thus establishing the material groundwork to combat marine bio-pollution.

**Figure 9:**
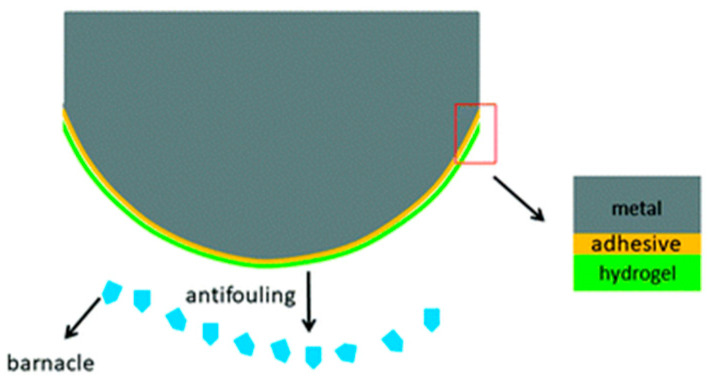
PVA–glycerol composite hydrogel for stainless-steel substrates [99].

Within the sphere of marine antifouling, vessels traverse the sea, and to harness the attributes of hydrogel for ship coatings, the issue of the hydrogel’s arduous adhesion to solid surfaces necessitates resolution. Figure 9 illustrates Zhu et al.’s [99] examination of the antifouling capabilities of composite hydrogel and the adherence properties of the PVA–glycerol composite hydrogel on a stainless-steel substrate employing a polyallylamine hydrochloride hydrogel and α-cyanoacrylic acid ethyl ester as the adhesive. Experimental findings elucidate that the PVA–glycerol composite hydrogel displayed commendable mechanical properties and robust adhesion to the stainless-steel substrate in the course of utilization, and the hydrogel coating curbed the settlement of white ridge barnacles effectively. Moreover, Ghani et al. [100] synthesized four hydrogel coatings—hydroxyethylcellulose gel (HEG), hydroxyethyl acrylate copolymer (HAAC), human acellular amniotic membrane (HAAM), and PVA-MMA—by co-polymerizing three monomers, acrylamide (AAM), acrylic acid, and ethylene glycol (EG), with PVA and methyl methacrylate (MMA) through semi-intermittent emulsion polymerization, and they scrutinized the effects of hydrogel coatings on equilibrium water content and adhesive potency. The outcomes indicated that PVA combined with acrylic acid possessed the highest adhesive strength to carbon steel in its dry state.

### 3.2. PEG-Based Hydrogel Coatings

PEG along with its oligomers exhibits protein-resistant characteristics, which have found significant applications in diverse product developments, particularly in marine antifouling [101]. Owing to the pronounced polarity of the oxygen atom within its main chain structure, this material is distinctly hydrophilic [102]. The principal process in fabricating PEG-based hydrogels centers on the modification of PEG’s hydroxyl terminus and the integration of a multitude of regulated chemical cross-linking functional clusters. Augmenting hydrogel coatings’ mechanical properties and addressing their low hardness and vulnerability to damage carry substantial implications for marine antifouling [103]. The toughness and longevity of the hydrogel coatings have a direct impact on their effectiveness and persistence within marine settings. Enhancing the coatings’ resilience, impact resistance, compression resistance, and adherence can efficaciously safeguard maritime structures’ surfaces, curb maintenance expenses, prolong operational lifespan, and conserve marine environments and ecosystems.

**Figure 10:**
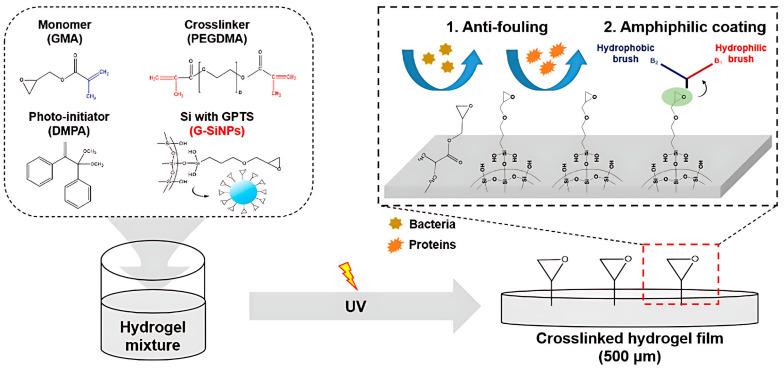
Chemical reaction principle for the preparation of PS-PEG hydrogels [104].

**Figure 11:**
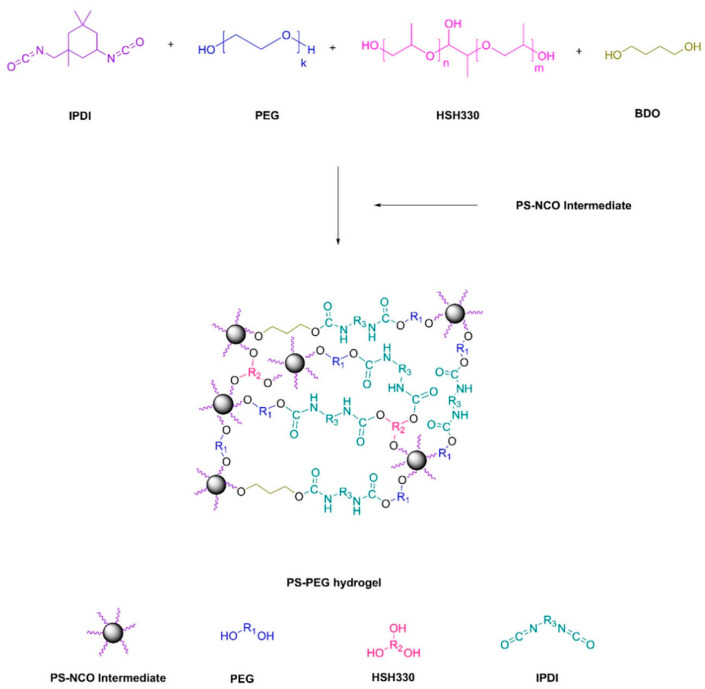
GPTS—G-SiNPs hydrogel film preparation process [105].

As depicted in Figure 10, Zhang et al. [104] implemented a styrene soapless emulsion polymerization to synthesise polystyrene microspheres exhibiting a core–shell structure, employing styrene, hydroxyethyl methacrylate, and poly(ethylene glycol) methyl ether methacrylate as monomers. Following this, PEG composite hydrogel was prepared using PEG hydrogel as a matrix. It was evident that the inclusion of an adequate number of polystyrene microspheres significantly bolstered the mechanical characteristics of the PEG composite hydrogel. The composite hydrogel’s maximum compressive stress was 5.83 MPa at a mass fraction of polystyrene microspheres of 14.2%, the relative compression ratio was 161.96%, and the compressive elastic modulus equaled 73.7 kPa. As illustrated in Figure 11, Park et al. [105] utilized poly(ethylene glycol) methacrylate as the cross-linking agent, glycidyl methacrylate as the monomer, and 2,2-dimethoxy-2-phenylethanone as the photoinitiator to synthesize PEG hydrogels. Subsequently, commercially accessible silica nanoparticles (G-SiNPs) underwent modification utilizing 3-glycidoxypropyltrimethoxysilane encompassing epoxy groups. These modified G-SiNPs were integrated into the PEG hydrogels. Observations revealed that these adapted silica nanoparticles significantly enhanced the surface mechanical properties of the hydrogel coatings, thereby resolving the issues associated with their low hardness and susceptibility to damage. Wanka et al. [106] engaged in the covalent coupling of dendritic polyglycerol (PG) with hydroxyethyl methacrylate (HEMA) through anion-catalyzed ring-opening polymerization, leading to the production of dendritic PG-HEMA, which encompassed four PG repeating units. This was followed by the preparation of a methacrylate monomer coating via grafting. This product was subsequently amalgamated with commercially procurable hydrophilic monomers—HEMA, polyethylene glycol methacrylate, and polyethylene glycol methacrylate—to perform dynamic accumulation experiments and dynamic short-term antifouling tests in marine settings. The experimental outcomes demonstrated that nearly all glycol derivatives depicted efficient inhibition in marine biological sedimentation experiments. Further, the enhancement of the anti-adhesion properties of hydrogels has the potential to provide protection for marine structures surfaces, curb marine pollution, and preserve the marine ecological equilibrium.

Despite PEG, poly(2-hydroxyethyl methacrylate) (pHEMA), and dextran being part of the roster of hydrophilic polymers, PEG is frequently hailed as the benchmark for antifouling substances due to its limited protein engagement and efficient hydration through hydrogen bonding [107]. However, recent findings suggest that PEG may not be the most favourable choice for antifouling material. Predominantly, these antifouling properties that PEG materials possess are stringently confined to the application environment [108,109]. Furthermore, PEG demonstrates susceptibility to oxidation when situated in biological media, which consequently leads to a considerable amount of protein adsorption [110]. Lastly, the affinity of PEG materials with proteins is fairly weak [111,112]. Shao et al. [113] explored the influence of amphiphilic carboxybetaine (CB) groups and nonionic oligoethylene glycol (OEG) groups on the hydrophobic interactions between two nonpolar plates, utilizing robust simulations of tempered shape dynamics. The findings suggest that the hydrophobic interactions under CB solution and water conditions remain identical, while these interactions are attenuated in OEG solution, with new associative states becoming evident. This implies that OEG’s intrinsic hydrophobic properties can meddle with the hydrophobic connections between the two nonpolar plates. Furthermore, recent research has corroborated the presence of PEG antibodies, whereas amphiphilic ionic materials demonstrate minimal immunogenicity [114,115,116]. These identified problems underscore the necessity for more effective antifouling materials.

### 3.3. Hydrogel-Organic Resin Hybrid Network Coating

Comprising both the hydrophilic and hydrophobic components of an organic resin, hydrogel-organic resin hybrid network coatings form a hydrogel layer on a solid surface through the absorption and leaching process of polymers from the hydrophilic component. Such hybrid coatings have a significant role in marine antifouling, harnessing the benefits of both hydrogels and organic resins. These modifications enhance the material’s durability against the marine environment, extending its lifespan and offering robust antifouling and anti-corrosion properties. These attributes subsequently lead to reductions in maintenance expenses and cleaning requirements.

**Figure 12:**
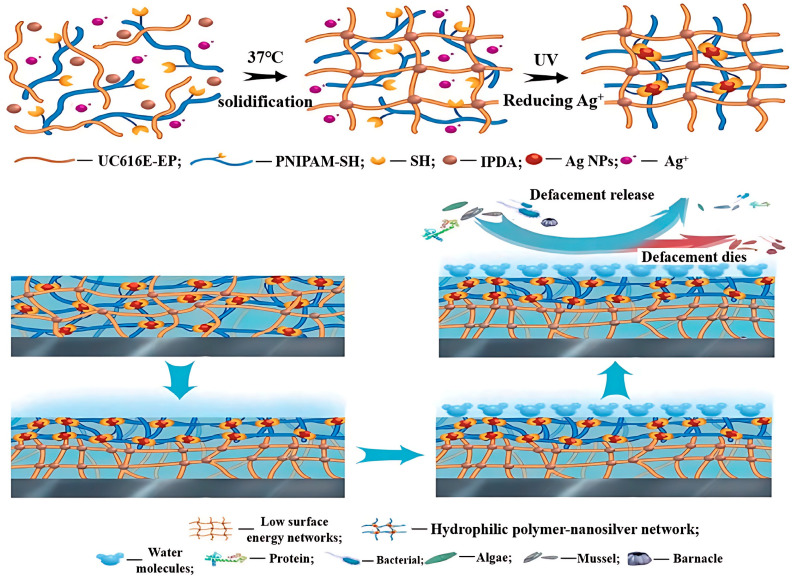
Preparation process and antifouling mechanism of amphiphilic coating of epoxy silicone/PNIPAM-SH hydrogel [43].

Lu et al. [43] crafted an antifouling coating constituting a hybrid network of hydrophobic epoxy organosilicon resin interlaced with a hydrophilic hydrogel. This is demonstrated in Figure 12, where the hydrogel network, created by silver nanoparticles (Ag NPs), effectively cross-links chains of mercapto-isopropyl acrylamide (PNIPAM-SH) polymers. The coating performs optimally when the Ag NPs and PNIPAM-SH contents are, respectively, 1% and 5%. The epoxy silicone resin network delivers outstanding mechanical properties and adhesive strength, boasting a tensile strength of up to 1.2 MPa and achieving a top-ranking adhesion value of 5B, as per the ASTMD 3359 standard. The Ag NP hydrogel network provides impressive antifouling properties. Lab test results confirm the coating’s marked resistance against contamination of proteins, bacteria, and microbial matter. The underlying antifouling mechanism involves the destruction of marine organisms attaching to the coating in the marine environment, driven by the Ag NPs within the hydrogel network.

**Figure 13:**
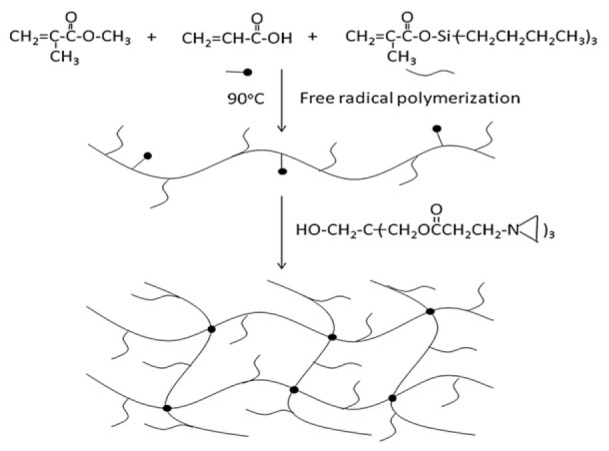
Self-polishing diagram of the novel coating immersed in seawater and schematic diagram of hydrogel generation and self-peeling [15].

**Figure 14:**
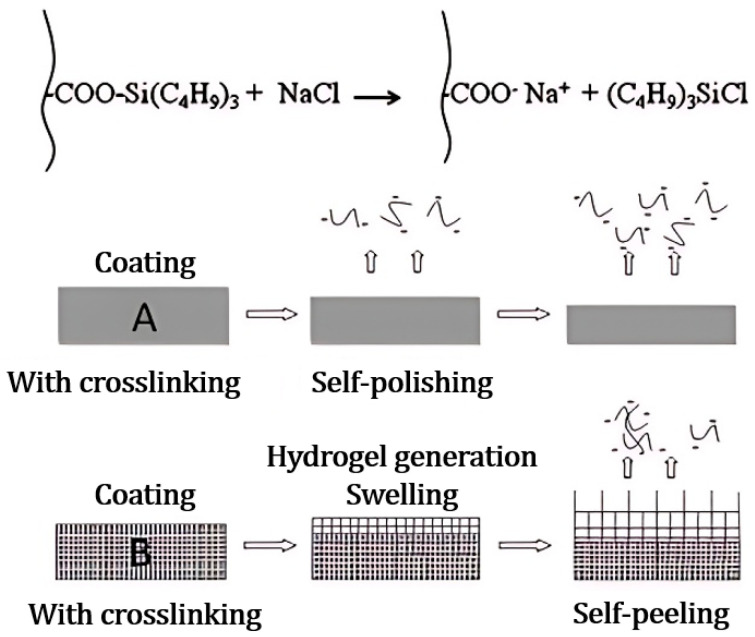
Schematic diagram of resin synthesis and coating preparation [15].

As demonstrated in Figure 13 and Figure 14, Xie et al. [15] developed a self-polishing coating using a ternary co-polymerization process involving methyl methacrylate (MMA), acrylic acid (AA), and trimethylsilyl methacrylate (TBSM) to form an acrylic resin, subsequently employing a trifunctional aziridinium agent for cross-linking. Post-immersion in seawater, hydrolysis of TBSM yields anionic and hydrophilic carboxyl groups, prompting the coating’s outer surface to spontaneously generate a soft, dynamically renewed thin layer of hydrogel. Further, a higher TBSM content offers improved antifouling performance, while the hydrophobic component of the bottom layer assures ample adhesion and mechanical strength. Following two-month seawater immersion, field tests on hanging boards verified that the coating remained largely intact and exhibited good anti-barnacle adhesion. The hydrogel’s hydrophilic component imparts an antifouling function to the hydrogel-organic resin hybrid network coating, while the organic resin’s hydrophobic component provides substantial adhesion and mechanical strength.

### 3.4. Dual-Network Hydrogel Coating

The limitations of mono-reticulated hydrogels, in particular their weak mechanical properties, fatigue resistance, and self-restoring healing properties, greatly restrict their practical uses. DN hydrogels, a type of developed self-healing hydrogel, show comparatively high mechanical performance, relating to their more robust network entanglement [117,118,119,120]. Typically, DN hydrogel designs incorporate two chemically cross-linked networks. Yet, recent developments have seen a minor number of physically/chemically cross-linked hybrid DN hydrogels that replace the first network’s covalent bonds with non-covalent ones [121,122,123,124,125]. Unlike a single-network hydrogel, a double-network hydrogel coating combines the qualities of two different hydrogels through the introduction of a double-layer network structure, improving the antifouling performance, mechanical properties, and anti-corrosion performance, to amplify the stability of the coating’s performance for marine antifouling applications.

The self-healing capability of DN hydrogels plays a pivotal role in marine antifouling. Maintaining a harmonious blend of robust mechanical attributes and regenerating features within these hydrogels persist as significant areas of exploration. Chen and their team constructed a dual-network (DN) gel, with the initial network constituting hydrogen-bonded cross-linked agar and the subsequent one comprising polyacrylamide (PAAm) linked through covalent bonds [117,126,127]. Furthermore, a variety of hybrid ionic/covalently cross-linked DN gels have been synthesized by researchers, with anionic polysaccharides (such as alginate, gellan gum, and carrageenan) serving as the initial ionic cross-linked network and PAAm as the secondary covalently cross-linked network. Despite these hybrid cross-linked DN gels exhibiting desirable strength and toughness, their self-repair remains slow and inefficient, credited to the sluggish dispersal of chains and the unalterable fracturing of covalent connections within the chemically interlinked structure [118,128,129,130]. To boost their self-repair competency, Gong et al. [83] conceived and prepared a DN hydrogel with a distinctive mechanism. In this scenario, the functional building blocks from the secondary framework are diffused in a uniform pattern into the primary structure through the process of solubilization and dispersion. This lends the hydrogel exceptional mechanical properties—hardness and toughness—and a strength magnified by over 20-times compared to the single-network hydrogel that originally constituted it. Liu et al. [131] established a DN hydrogel by marrying the ionic cross-linked network of carrageenan and a polyacrylamide framework interconnected through covalent bonds, capitalizing on the thermally reversible transition behavior of the carrageenan sol/gel in water. After the severed samples underwent a specified storage period under temperatures exceeding the gel–sol transition threshold, a self-healing phenomenon was noticed. Notably, the samples showcased remarkable self-healing abilities and sound restorative properties, even in the presence of gaps within the hydrogel.

**Figure 15:**
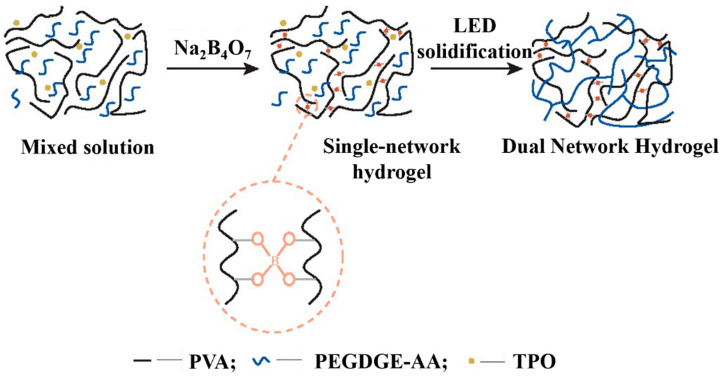
The preparation process of dual-network hydrogel [132].

As depicted in Figure 15, Hu et al. [132] utilized a one-pot method, blending epoxy acrylate with photoinitiators diphenyl (2,4,6-trimethylbenzoyl) phosphine oxide, PVA, and sodium tetraborate decahydrate. They consequently developed epoxy acrylate (PEGDGE-AA)/PVA bi-networked hydrogels for marine antifouling, taking advantage of light-emitting diode light curing technology, and evaluated the hydrogel’s mechanical and antifouling performance. The empirical results indicated that the DN hydrogel’s tensile attributes were considerably superior to PVA single-network hydrogel, with DN hydrogel significantly impeding the attachment of Rhodophyta minor crescents without manifesting toxicity. Meanwhile, Tang et al. [133] synthesized a DN hydrogel leveraging a dual-network structure of a gelatin framework coupled with the structure of poly-N hydroxyethyl acrylamide entirely physically cross-linked via hydrogen bonding. The hydrogel possesses robust mechanical properties, accelerated self-recovery, effective self-healing, and pronounced surface adhesion. Sun et al. [134] achieved a DN hydrogel with pervasive adhesion and high cyanocompatibility by blending vinylphosphate (VPA) into the network, founded on a DN hydrogel of agar and acrylamide composition while preserving its formidable strength and durability. Cellular studies illustrated that integrating an appropriate concentration of VPA promoted cell adhesion and proliferation, introducing a novel approach for the enhancement of the DN hydrogels’ functionality. Additionally, Yang et al. [135] explored a hetero-ionic-covalent DN hydrogel that retained impressive mechanical strength and resilience, as well as a good modulus of elasticity and tensile strength post-immersion in water. Through modification of the cross-linking duration, the density and rigidity of the chitosan ionic network can be controlled, thereby adjustably influencing the structure and mechanical integrity of the hydrogel.

In the grand scheme of things, DN hydrogel coatings, functioning as a marine antifouling coating with good comprehensive performance, bring to light specific strengths in antifouling efficiency, mechanical robustness, and anti-corrosion effectiveness [136]. However, associated challenges and shortcomings—most notably in the realms of preparation intricacy and stability—persist. This mandates continuous optimization and improvement in upcoming research and practical applications so as to maximize its potential utility in the marine antifouling domain [137].

### 3.5. Amphiphilic Ionic Hydrogel Coatings

Amphoteric ionic polyelectrolyte hydrogel embodies a spectrum of both anionic and cationic groups within its polymer network. These amphoteric ions exhibit strong water molecule attachments via ionic solvation, initiating repulsion and effectively thwarting protein absorption and the adherence of both bacterial and cellular elements. Indeed, this component serves a dual role: it not only provides antibacterial properties but also antifouling attributes, thereby hindering bacterial adhesion, thus positioning it among the class of hydrogel materials that hold vast potential for diverse applications [104,105].

**Figure 16:**
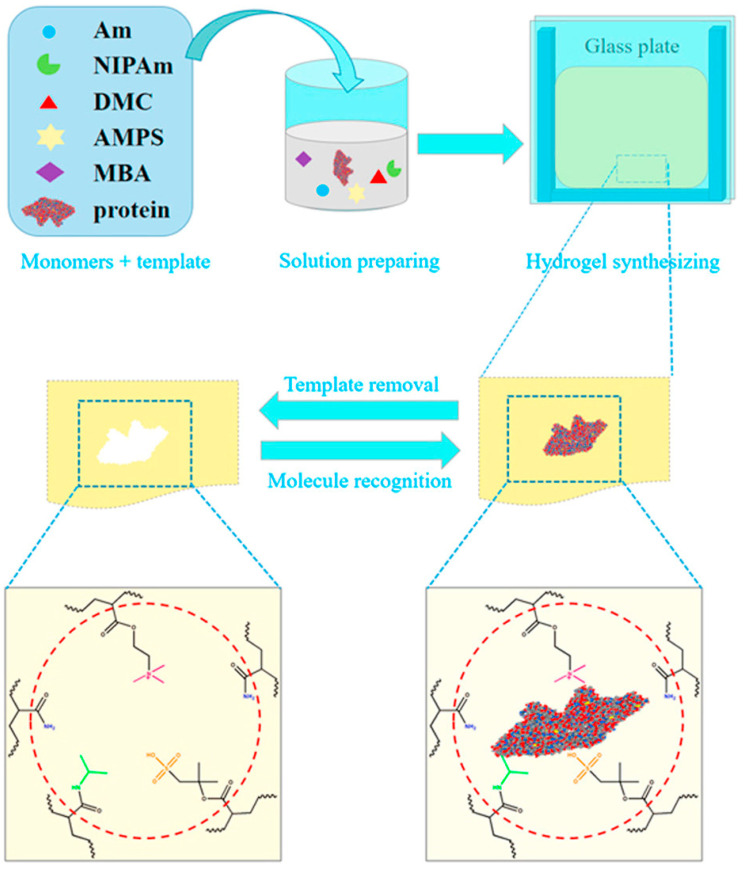
Ampholytic electrolyte hydrogel preparation process. The blue circle is acrylamide(Am), the green shape is N-isopropylacrylamide (NIPAm), the red triangle is [2-(methacryloyloxy)ethyl]trimethylammonium chloride(DMC), the golden five-pointed star is 2-acrylamido-2-methyl-1-propanesulfonic acid (AMPS), the purple prism is *N*,*N′*-methylenebisacrylamide (MBA), and the red and black dots are protein [143].

Amphiphilic ionic hydrogels are synthesized and capable of assembly via physical interactions, thus also termed as supramolecular hydrogels. In recent years, both amino acid derivatives and dipeptides have been recognized as constituents of these supramolecular hydrogels [130,138]. Hydrophobic interactions or hydrogen bonds serve as physical cross-linking agents in these self-assembled peptide hydrogels. Peptides of phenylalanine and its derivatives are suited for creating transparent hydrogels through self-assembly, attributed to the potent π-π stacking of aromatic groups [139,140]. Tsutsumi et al. [141] further incorporated urea bonds to cultivate a robust hydrogen bonding network, stabilizing the self-assembled structure. This approach eliminates potentially toxic small molecules during hydrogel formation, rendering it apt for applications requiring a pristine environment [141,142]. Such hydrogels contribute to maintaining cleanliness and stability in the marine environment, presenting more eco-friendly solutions and yielding numerous benefits to the marine ecosystem. As demonstrated in Figure 16, Ying et al. [143] synthesized an amphiphilic ionic electrolyte hydrogel using acrylamide as the structural monomer alongside N-isopropylacrylamide, [2-(methacryloyloxy)ethyl] trimethylammonium chloride, and 2-acrylamido-2-methyl-1-propanesulfonic acid as the functional monomers. Incorporating both positively and negatively charged acrylic monomers into the hydrogel facilitates specific reintegration and selective recognition of the material. Examining the molecular recognition properties and underlying mechanisms of amphiphilic electrolyte hydrogels aids in unraveling electrostatic interactions between charged biomolecules, adsorbates, as well as protein recognition. Owing to their distinct recombination and selective recognition capabilities, these hydrogels have utility in studies of electrostatic interactions and hold promise for applications in marine antifouling material development, notably in the recognition and elimination of specific biomolecules, thereby enhancing the performance and efficiency of these materials.

**Figure 17:**
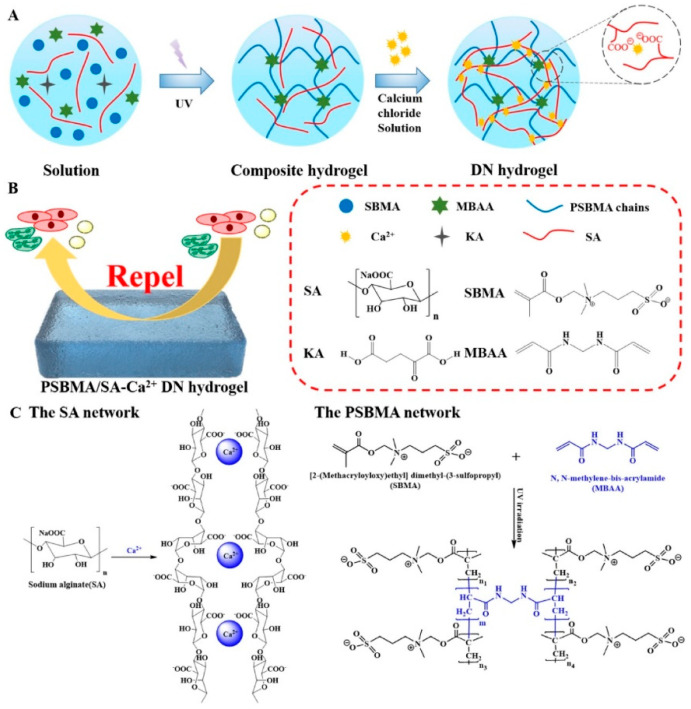
DN hydrogel should be listed as: (**A**) Preparation of PSBMA/SA-Ca^2+^DN hydrogels by the “one-pot method”. (**B**) Representation of the antifouling properties of DN hydrogels against algae (green), cells (red) and non-specific proteins (yellow). (**C**) Synthetic routes and formation of SA and PSBMA networks in DN hydrogels [144].

Advancements in amphiphilic ions continue to catalyze innovation in DN hydrogel production. As shown in Figure 17, Zhang et al. [144] prepared a novel DN hydrogel utilizing amphiphilic ion materials—sulfobetaine methacrylate and sodium alginate, a natural polysaccharide—as primary constituents. The poly(sulfobetaine methacrylate) network undergoes covalent cross-linking, while the sodium alginate network experiences Ca^2+^ ionic cross-linking. The dual polymer frameworks interpenetrate, and their hetero-cross-linked dual-network structure imparts exceptional mechanical properties to the DN hydrogel. The DN hydrogel network embodies numerous non-covalent interactions, such as ionic and hydrogen bonding, generally weaker than covalent bonding. Consequently, during deformation, these non-covalent bonds disrupt first, effectively dissipating energy, facilitating rapid self-healing, good fatigue resistance, and resistance against unspecific protein adsorption and microbial adhesion, such as cellular algae, thereby demonstrating remarkable antifouling properties. Long et al. [145] synthesized an amphiphilic electrolyte hydrogel anchored on electrostatic interactions occurring between imidazole and sulphonate groups. The hydrogen bonding within urea groups functions as sacrificial bonds, thereby enhancing the mechanical properties of the hydrogels. These hydrogels manifest notable self-healing attributes at room temperature because of reversible electrostatic and hydrogen bonding interactions. Hydrogels exhibiting such self-healing traits possess the capability to restore themselves following damages or abrasions incurred in the marine environment, which ultimately extends their lifespan. Harnessing this distinctive property of hydrogels in marine antifouling materials augments their durability, curtails maintenance expenses, and effectively deters marine pollutants from adhering and accumulating in the marine environment. This approach significantly contributes to preserving marine cleanliness and health.

The five types of hydrogel network coatings exhibit unique characteristics, imparting distinct advantages and limitations. PVA-based hydrogel coatings, recognized for their good biocompatibility and water solubility, proficiently curb biological attachment and contamination. Nonetheless, their durability and mechanical strength lag behind, warranting future improvements in these parameters through cross-linking methodology and bolstering mechanical attributes. In contrast, the PEG-based hydrogel coatings are lauded for their outstanding hydrophilicity and reduced protein adsorption, decreasing adhesion and protein contamination. Despite these benefits, their durability falls slightly short compared to other coatings, necessitating the exploration of durability-enhancing strategies such as hybridized network structure design. The hydrogel-organic resin hybrid network coating amalgamates hydrogel and organic resin virtues, offering weather resistance, antifouling, and mechanical properties. Even though suited for long-term maritime antifouling coatings, the performance stability can benefit from further research. Dual-network hydrogel coatings integrate two alternating overlap network structures, enhancing both antifouling and mechanical properties, an attribute beneficial for maritime antifouling but requiring design strengthening to broaden the application scope and reliability. Amphiphilic ionic hydrogel coatings, appreciated for their binary ionic attributes, boast balanced surface charges and superb resistance to bio-adhesion and protein adsorption. As such, immediate research priority lies in scrutinizing opposing ionic property combinations to optimize coating performance and stability.

## 4. Conclusions

### 4.1. Discussion of the Future

Mitigating the subpar mechanical attributes of hydrogels has led to the recent emergence of a new variety of hydrogel materials, including PVA-based hydrogel coatings, PEG-based hydrogel coatings, hydrogel-organic resin hybrid network coatings, dual-network hydrogel coatings, and amphiphilic ionic hydrogel coatings. These are catalyzed by diverse cross-linking mechanisms and assembled functional monomers. The PVA-based hydrogel coating offers good biocompatibility and water solubility but is let down by its subpar durability and mechanical strength. The PEG-based hydrogel coating stands out due to its good hydrophilicity and low protein adsorption properties, although it is less durable. The hydrogel-organic resin hybrid network coating excels in weather resistance, antifouling, and mechanical properties, despite requiring further study to evaluate the stability of its performance. The dual-network hydrogel coatings can heighten the antifouling and mechanical properties of the coating, but its design would still need reinforcement to improve the coating’s application range and reliability. Lastly, the amphiphilic ionic hydrogel coatings, harboring bipartite ionic characteristics, perform well against bio-adhesion and protein adsorption, thanks to their balanced surface charge. However, collective optimization efforts are necessary to improve its performance and stability.

The continuous refinement and research into hydrogels underscore their burgeoning potential in marine antifouling applications, thereby offering innovative solutions for managing marine environmental pollution. However, hydrogels also face some challenges and limitations in marine antifouling applications; although the soft and wetting properties of hydrogels help in antifouling, they also make them deficient in adhesion and mechanical properties, which limits their use in some applications that require high strength and durability. Although hydrogels have good antifouling properties, their antifouling capability needs to be further strengthened, especially in the face of the complex marine biofouling environment, where a single hydrogel material may not be able to provide durable and broad-spectrum antifouling effects; traditional antifouling coatings may be harmful to the marine ecosystem, so the development of environmentally friendly hydrogel antifouling materials has become the focus of research. However, this also brings challenges in terms of cost and preparation process; hydrogels may be affected by biodegradation and physical abrasion in the long-term marine environment, thus affecting their durability, so improving the durability of hydrogels is the key to realizing their application in marine antifouling; hydrogels need to have the ability to fight against fouling by a wide range of marine organisms, including bacteria, algae, barnacles, and so on. However, the broad spectrum of antifouling hydrogels still needs to be improved; environmentally friendly, low-cost methods that can be mass-produced are more attractive to the marine industry, so how to ensure the antifouling performance of hydrogels while reducing costs and achieving mass production are issues that need to be resolved for the application of hydrogels in the field of marine antifouling.

### 4.2. Conclusions of Future Research

Boasting good physicochemical properties, hydrogels present extensive potential applications in marine antifouling arenas. An evaluation of current hydrogel utilizations in these spaces suggests that hydrogel’s remarkable attributes provide a robust material foundation for marine antifouling. Primarily, hydrogels exhibit proficient adsorption characteristics and permeability, capably adsorbing and entrapping oceanic pollutants such as oils and microplastics, thereby alleviating marine ecosystem pollution. Further, hydrogels possess reliable controlled-release abilities, facilitating their design as materials with custom functionalities and release rates, allowing for the discharge of adsorbed and immobilized environmentally friendly detergents or biofouling agents for the targeted intercession and safeguarding of the marine environment.

Future research can improve the mechanical properties and self-healing ability of hydrogel antifouling materials by changing the hydrogel network structure, cross-linking, and adding nanomaterials. The antifouling ability of hydrogel needs to be further strengthened. Researchers can improve the antifouling performance of hydrogel polymers by grafting or copolymerizing monomers with the antifouling ability or special functions onto hydrogel polymers. In addition, the bionic antifouling strategy is an important research direction in the future, for example, to mimic the surface properties of natural organisms (e.g., lotus leaf, shark skin, etc.) to develop antifouling surfaces with micro-nano structures. The development of multifunctional biomimetic antifouling coatings, combined with the synergistic advantages of multiple antifouling strategies, can overcome the limitations of single-function antifouling strategies and significantly improve the durability and antifouling capacity. In the future, natural antifouling agents or their synthetic analogs can be extracted from a wide range of organisms based on the principle of ‘from nature to nature’, and the chemical composition and structure of these natural antifouling agents can also provide a reference for the development of new types of antifouling coatings for the oceans. In the future, the development of environmentally friendly and non-toxic antifouling materials can be enhanced along with the awareness of marine environmental protection. Future research on hydrogel coatings with good drag reduction effects should be carried out to reduce the operating resistance of marine equipment and improve energy efficiency and develop hydrogels with good anti-protein adsorption and anti-bacterial properties to reduce the occurrence of marine biofouling, improving the adhesive properties of hydrogels on the surface of different substrates to ensure the long-term stability of antifouling coatings. Future research and the development of environmentally friendly, low-cost methods that can be mass-produced to meet the needs of the marine industry must be conducted.

Future explorations can hone in on optimizing structural design, enhancing material properties of assorted hydrogel coatings, assessing their long-term stability within the marine milieu, and evaluating the impact and efficacy of their practical implementation. Such focused efforts are bound to unlock greater possibilities and resolutions for deploying hydrogel coatings in marine antifouling applications.

## Figures and Tables

**Table 1:** Classification of hydrogels.

Hydrogel	Polymers	Functional Characteristics
Natural source hydrogel	Capsaicin N-(4-hydroxy-3-methoxybenzyl)acrylamide	Good hydrophilicity, high mechanical strength, low swelling rate, good antifouling effect [77]
Synthetic hydrogel	Polyvinyl alcohol (PVA), polyacrylamide (PAM), polyacrylic acid (PAA), polyethylene glycol (PEG), polyvinylpyrrolidone (PVP)	Good chemical stability, strong mechanical properties, easy to prepare, good antifouling properties [78]
Hybrid hydrogel	Synthetic polymer	Low treatment cost, meets a wide range of water treatment scenarios [79]

## Data Availability

The data that support the findings of this study are available from the corresponding author upon request.
